# Optimizing bacterial DNA extraction in urine

**DOI:** 10.1371/journal.pone.0222962

**Published:** 2019-09-24

**Authors:** Matthew M. Munch, Laura C. Chambers, Lisa E. Manhart, Dan Domogala, Anthony Lopez, David N. Fredricks, Sujatha Srinivasan

**Affiliations:** 1 Vaccine and Infectious Disease Division, Fred Hutchinson Cancer Research Center, Seattle, Washington, United states of America; 2 Department of Epidemiology, University of Washington, Seattle, Washington, United states of America; 3 Department of Global Health, University of Washington, Seattle, Washington, United states of America; 4 Department of Medicine, University of Washington, Seattle, Washington, United states of America; 5 Department of Microbiology, University of Washington, Seattle, Washington, United states of America; Weill Cornell Medicine, UNITED STATES

## Abstract

Urine is an acceptable, non-invasive sample for investigating the human urogenital microbiota and for the diagnosis of sexually transmitted infections. However, low quantities of bacterial DNA and PCR inhibitors in urine may prevent efficient PCR amplification for molecular detection of bacteria. Furthermore, cold temperatures used to preserve DNA and bacteria in urine can promote precipitation of crystals that interfere with DNA extraction. Saline, Dulbecco’s Phosphate Buffered Saline, or Tris-EDTA buffer were added to urine from adult men to determine if crystal precipitation could be reversed without heating samples beyond ambient temperature. Total bacterial DNA concentrations and PCR inhibition were measured using quantitative PCR assays to compare DNA yields with and without buffer addition. Dissolution of crystals with Tris-EDTA prior to urine centrifugation was most effective in increasing bacterial DNA recovery and reducing PCR inhibition. DNA recovery using Tris-EDTA was further tested by spiking urine with DNA from bacterial isolates and median concentrations of *Lactobacillus jensenii* and *Escherichia coli* 16S rRNA gene copies were found to be higher in urine processed with Tris-EDTA. Maximizing bacterial DNA yield from urine may facilitate more accurate assessment of bacterial populations and increase detection of specific bacteria in the genital tract.

## Introduction

Cultivation and molecular approaches have demonstrated that human urine commonly harbors genital tract bacterial commensals and pathogens [1–8]. Urine samples are frequently used to detect sexually transmitted microbes such as *Chlamydia trachomatis*, *Neisseria gonorrhoeae*, *Mycoplasma genitalium* and *Trichomonas vaginalis* using nucleic acid amplification methods [9–13]. Emerging molecular techniques such as nanopore-based metagenomic sequencing are being considered for clinical applications, including testing for bacterial pathogens in urine samples [14]. The role of the urinary tract microbiota in relation to outcomes of clinical importance, such as urinary tract infections [15–21], urinary incontinence [21–27], interstitial cystitis [28,29], bacterial vaginosis [30], urethritis [31], cancer [32–36], type 2 diabetes [17,37,38], kidney disease [39] and kidney transplants [40,41], is an area of active investigation. Collection of non-invasive urine samples is more acceptable to men than collection of urethral swabs [42] and is a convenient alternative to urethral swabs in studies of the male urethral microbiota [43]. However, optimal methods for processing urine samples for PCR-based studies have not been defined [44].

Concentrations of bacteria in urine are typically low compared to other human body sites, and urine contains PCR inhibitors such as urea, beta-human chorionic gonadotropin, and crystals that can impede bacterial DNA detection [45,46]. One approach to circumvent low DNA concentrations is to perform DNA extraction from large volumes of urine (> 30 mL) to increase DNA yield. However, many types of crystals such as calcium oxalate, uric acid, and amorphous phosphate or urate crystals can precipitate out of solution at the low temperatures commonly used to store and preserve urine [47–49]. On centrifugation of urine to concentrate bacteria, these crystals form large pellets that can interfere with DNA extraction using commercially available kits and using larger urine volumes can exacerbate this problem further [49]. We have noted lower DNA yields and higher rates of PCR inhibition from urine samples with large pellets, and others have noted decreased DNA yields and lower DNA quality when crystals are present in pellets [49]. Urine temperature, pH, and concentrations of solutes all affect crystal formation [47,48,50]. Urine pH, which ranges from 4.5–8, has a large impact on crystal formation and can determine which crystals form [51]. For example, uric acid crystals form in acidic urine (typically pH <5.8), whereas calcium phosphate and amorphous phosphate crystals form in neutral or alkaline urine [47,48,50]. Conversely, calcium oxalate crystals form in a wide pH range, and are more dependent on urine calcium and oxalate concentrations [50]. Many of these crystals can be dissolved by heating urine specimens [47], but there is potential for DNA degradation or growth of bacteria with this approach, possibly biasing results. We sought to develop a method for reversing crystal precipitation in urine while preserving bacterial DNA and minimizing opportunities for bacterial growth, resulting in higher yield DNA extractions for molecular applications. A secondary goal was to develop a method that was cheap, efficient, and easily adaptable for high-throughput applications.

## Methods

### Sample collection

Urine specimens were collected from men with and without nongonococcal urethritis (NGU) attending the Public Health—Seattle & King County Sexually Transmitted Diseases Clinic in Seattle, Washington from February 2015 to September 2017 for a study investigating NGU in men [52]. The Institutional Review Boards at the University of Washington and Fred Hutchinson Cancer Research Center approved the protocols used in the study and the research was conducted according to all relevant guidelines and regulations. All men enrolled in the study provided written informed consent. Participants were ≥16 years of age, had exclusive male or female partners in the last year and had sex in the past two months. Men who had both male and female partners, no sex in the past 60 days, antibiotics in the past 30 days, known urethral contact to *Neisseria gonorrhoeae* (NG) or were diagnosed with NG by Gram stain or nucleic acid amplification testing were not eligible for the study. Men were enrolled into either a cross-sectional or cohort study and men in the cohort study provided more than one urine specimen at different time points. At all study visits, men underwent a standard genital examination and collected 30–45 mL first void urine and a urethral swab specimen for Gram staining. All men also completed a computer-assisted self-interview that collected sociobehavioral and clinical data. The urethral exudates were Gram stained to enumerate polymorphonuclear neutrophils (PMNs) and examine for the presence of Gram-negative intracellular diplococci that are indicative of NG. Urine was tested for NG and *Chlamydia trachomatis* (CT) using the Aptima Combo 2 Assay and *Mycoplasma genitalium* (MG) using the Aptima Assay with analyte-specific reagents (Hologic, Marlborough, MA). In this study, we defined NGU as having either urethral symptoms (urethral discharge, dysuria, or other urethral symptoms) or visible discharge on examination with ≥5 PMNs per high power field in the absence of NG. Men without NGU did not have urethral symptoms or urethral discharge and had <5 PMNs/HPF. Urine samples were stored at 4°C for 1–3 days prior to processing in the laboratory.

### Optimization of crystal dissolution

When testing for optimal approaches to process urine, 12 urine samples (33–37.5 mL in volume) from 12 men with noticeable crystal precipitation after refrigeration were split into two aliquots of equal volume, with the first aliquot serving as the pre-treatment sample and the other aliquot diluted with buffer serving as the post-treatment sample. Filtered 0.9% saline (2–15 mL) (AirLife Modudose, CareFusion, San Diego, CA), 1X Dulbecco’s Phosphate Buffered Saline (DPBS) (3–15 mL) (Gibco, Grand Island, NY), or molecular grade 1M Tris-ethylenediaminetetraacetate (EDTA) (0.5–1.25 mL) (Sigma Aldrich, St. Louis, MO) were each added separately to four different urine samples. The buffers were chosen due to their low cost, wide availability and routine use in clinical labs, as well as their isotonic nature with bacterial cells which would limit any cell lysis and loss of DNA. Additionally, DPBS and Tris-EDTA are buffered at a higher pH than urine, which may help in dissolution of crystals that form at lower pH such as uric acid [48,50]. We further postulated that EDTA may act as a chelating agent to reduce concentrations of Ca^2+^ in urine which would help reduce formation of calcium oxalate crystals [53]. Buffers were added in 1 mL increments (saline, DPBS) or 0.25 mL increments (Tris-EDTA) until crystal dissolution was achieved, and urine samples were brought to room temperature if necessary for complete dissolution of crystals. The urine was centrifuged post-treatment with buffers to concentrate the bacteria at 3645 × *g* at 4°C for 10 minutes, the supernatant discarded, and the urine pellet frozen at -80°C. Urine pH was measured before and after buffer addition with pH strips (mColorpHast^™^ pH Test Strips, EMD Millipore, Darmstadt, Germany). Mean log10 concentrations of bacterial DNA in pre- and post-treatment samples for each buffer were compared using a paired t-test.

### Spiking with DNA from bacterial cultures

An additional four urine specimens with crystal precipitation collected from four men were spiked with known quantities of *Escherichia coli* and *Lactobacillus jensenii* cells grown in culture to determine recovery rates of species-specific bacterial DNA using quantitative PCR (qPCR) with and without Tris-EDTA addition to urine. *E*. *coli* colonies were isolated on Luria Broth (LB) agar plates (Fisher Scientific, Waltham, MA) and grown aerobically in LB broth (Fisher Scientific, Waltham, MA). *L*. *jensenii* colonies were isolated on Brucella Blood Agar plates (Hardy Diagnostics, Santa Maria, CA) and grown aerobically in De Man, Rogosa and Sharpe (MRS) broth (Becton Dickinson and Company, Franklin Lakes, NJ). Colony Forming Units (CFU) of each culture were compared to Optical Density (OD) measurements with a spectrophotometer to estimate concentrations of bacteria in liquid cultures.

Urine specimens were divided into four aliquots of equal volume. Two aliquots were spiked to a final target concentration of 1×10^6^ CFUs/mL urine each with both *E*. *coli* and *L*. *jensenii* cultures, while the remaining two aliquots were not spiked with bacterial cultures. One culture-spiked aliquot of urine and one non-spiked aliquot were processed with addition of 10% by volume Tris-EDTA. The other aliquots were not treated with Tris-EDTA. Additionally, a positive control was employed to determine the maximum DNA yield, wherein equivalent quantities of both *E*. *coli* and *L*. *jensenii* cells were subjected to DNA extraction in the absence of urine. Comparisons of log10 concentrations of bacterial DNA between the groups were made using the paired t-test. All urine aliquots were pelleted and frozen as previously described.

### Application of optimized approach to clinical samples

The optimized protocol of 10% addition by volume of 1M Tris-EDTA was applied to additional male urine specimens (n = 998 from 273 men) with urine sample volumes ranging from 30–42 mL (median = 36 mL). This set of urine samples was processed as described previously, but no pre- and post-treatment comparisons were performed. We estimated the association between NGU and log10 concentration of bacterial DNA using generalized estimating equations, specifying a Gaussian model, an identity link function, robust standard errors, and an exchangeable covariance matrix.

### Molecular methods

DNA was extracted from urine pellets using the BiOstic Bacteremia DNA Isolation Kit (Mobio, Carlsbad, CA). DNA was eluted in 120 μL of half CB5 buffer and half-filtered 0.2X Tris-EDTA. Sham extraction controls were included to monitor for potential contamination during processing and DNA extraction of urine pellets. PCR inhibition was monitored using an internal amplification control (IAC) qPCR assay [54] and samples were considered inhibited if delayed by ≥ 2.0 cycles. Bacterial DNA concentrations were measured using a TaqMan based qPCR assay targeting a portion of the 16S rRNA gene [55] with a lower limit of detection of 5 gene copies/μL DNA. *E*. *coli* DNA concentrations were measured with a TaqMan based qPCR assay targeting the *E*. *coli* 16S rRNA gene with primers (47F 5ʹ-GAACGGTAACAGGAAKCAGCT-3ʹ and 194R 5ʹ- ATCCGATGGCAAGAGGCC -3ʹ) and hydrolysis probe (5′-FAM-CGCATAACGTCGCAAGACCAAAGAGG-TAMRA-3′). Reactions underwent 45 cycles of amplification on the Applied Biosystems StepOnePlus Real-Time PCR System with a 95°C melt for 15 seconds and 65°C anneal/extension for 1 minute. Core reagents were supplied by Applied Biosystems (Carlsbad, CA) and master mixes contained buffer A (1X), deoxynucleotide triphosphates (1 mM), magnesium (3 mM), AmpErase uracil-N-glycosylase (0.05 U) and AmpliTaq Gold polymerase (1.0 U) per reaction. Primers were added at 0.8 μM per reaction and probe at 150 nM per reaction. *E*. *coli* 16S rRNA gene plasmid standards were run for each reaction with a lower limit of detection of 5 gene copies/μL DNA. *L*. *jensenii* DNA concentrations were measured using a TaqMan based qPCR assay targeting the 16S rRNA gene [56] with a lower limit of detection of 1.25 gene copies/μL DNA.

## Results

### Optimization of crystal dissolution

**Fig 1:**
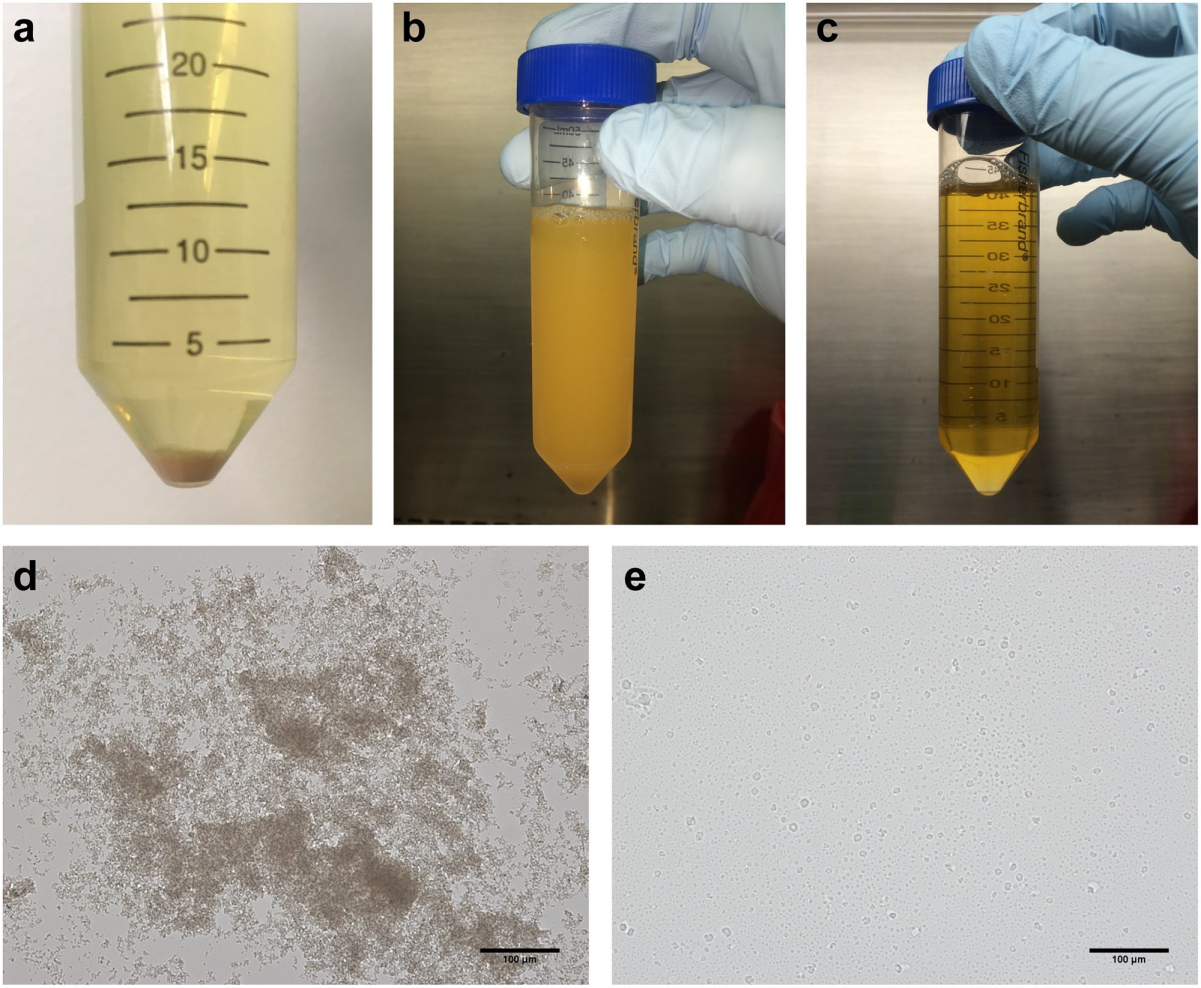
Images and microscopy of urine crystal precipitation and dissolution. (a) Pellet from refrigerated urine sample with crystal precipitation. After centrifugation, pellet is pink in appearance. (b) Refrigerated urine sample with crystal precipitation before and (c) three minutes after Tris-EDTA addition. (d) Microscopic view (20X) of crystals from urine pellet without and (e) with Tris-EDTA addition.

**Fig 2:**
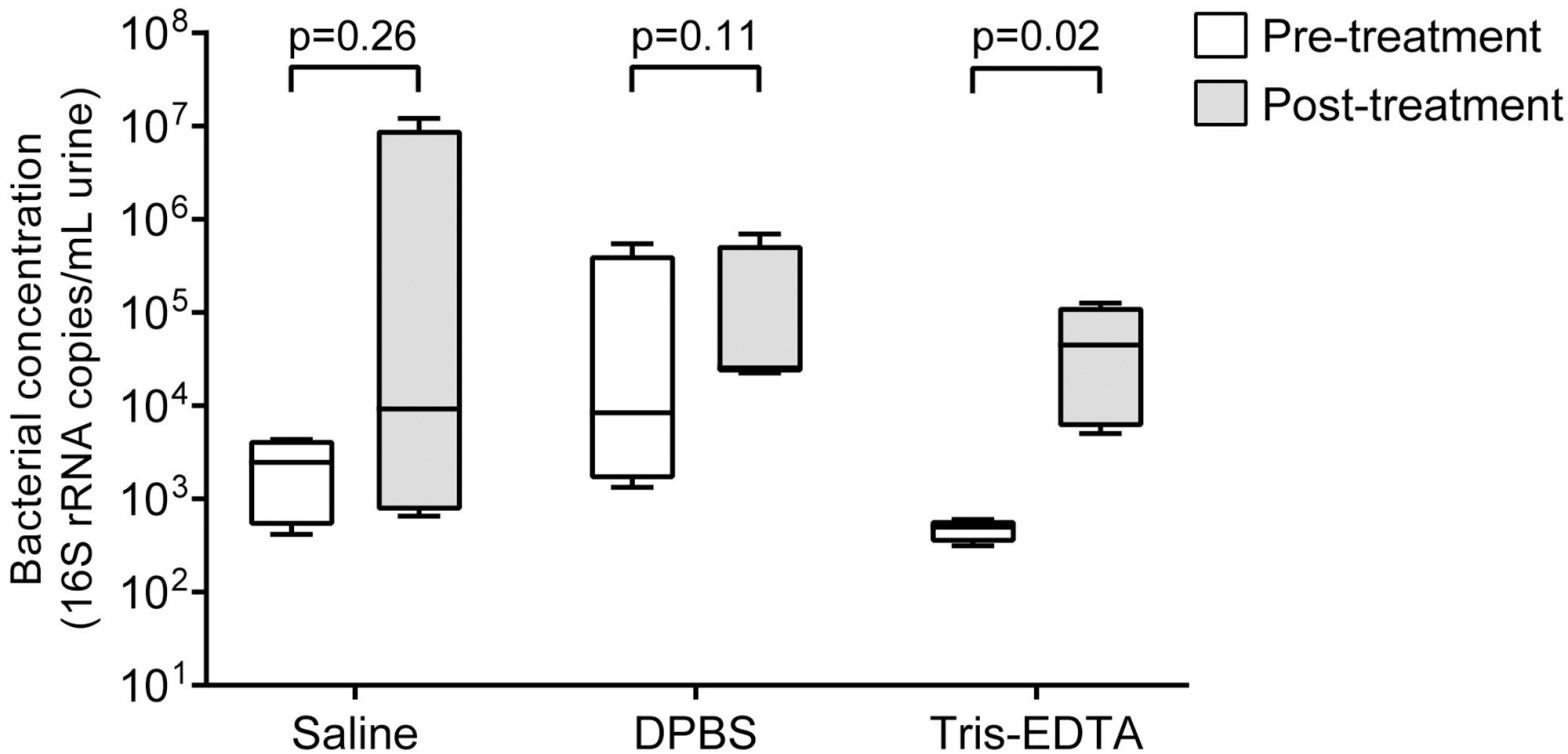
Bacterial DNA concentrations in urine samples before and after treatment with saline, DPBS, or Tris-EDTA. Data shown as box and whisker plots with whiskers representing maximum and minimum values (16S rRNA gene copies/mL of urine). Lines within each box represent median values. Mean bacterial concentrations in post-treatment samples treated with Tris-EDTA were found to be significantly higher.

The following metrics were used to evaluate each approach: visual disappearance of crystals in urine, volume of buffer added, shifts in pH, DNA yields, and absence or reduction of PCR inhibition. Addition of saline, DPBS, and Tris-EDTA dissolved precipitated crystals in each urine sample, but required varying amounts of buffer for complete dissolution, with Tris-EDTA requiring the least amount of buffer by volume (Fig 1 and Table 1). Urine pH was not impacted by the addition of saline or DPBS but was higher after the addition of Tris-EDTA (Table 1). Only aliquots treated with Tris-EDTA demonstrated mean post-treatment bacterial DNA quantities that were significantly higher than quantities in pre-treatment aliquots (mean 4.98×10^2^ copies/mL pre vs. 4.47×10^4^ copies/mL post, p = 0.02). There were no significant differences in aliquots treated with either saline (2.49×10^3^ vs. 9.22×10^3^, p = 0.26) or DPBS (8.43×10^3^ vs. 2.54 ×10^4^, p = 0.11) (Fig 2). The 16S rRNA gene copies in blank buffer samples were at or below the detection threshold of 20 copies/mL. PCR inhibition was reduced in three samples with addition of saline or Tris-EDTA, but not DPBS. PCR inhibition was not detected in any other samples.

**Table 1:** Volume of buffer required to dissolve urine crystals and urine pH pre- and post-treatment.

Sample ID	Buffer	Volume Buffer Required (mL)	Percent Buffer Required (v/v)*	Pre-Treatment Urine pH	Post-Treatment Urine pH
Sample 1	Saline	2.0	12.1	5.5	5.5
Sample 2		15.0	88.2	5.5	5.5
Sample 3		5.0	27.0	6.5	6.5
Sample 4		8.0	44.4	5.5	5.5
Sample 5	DBPS	4.0	24.2	5.5	5.5
Sample 6		5.0	29.4	5.3	5.3
Sample 7		15.0	85.7	5.5	5.5
Sample 8		3.0	16.7	5.5	5.5
Sample 9	Tris-EDTA	0.5	2.7	5.5	6.5
Sample 10		0.75	4.2	5.5	6.1
Sample 11		1.0	5.6	5.3	6.5
Sample 12		1.25	6.7	5.3	6.5

*v/v indicates volume/volume.

### Spiking with bacterial cultures

**Fig 3:**
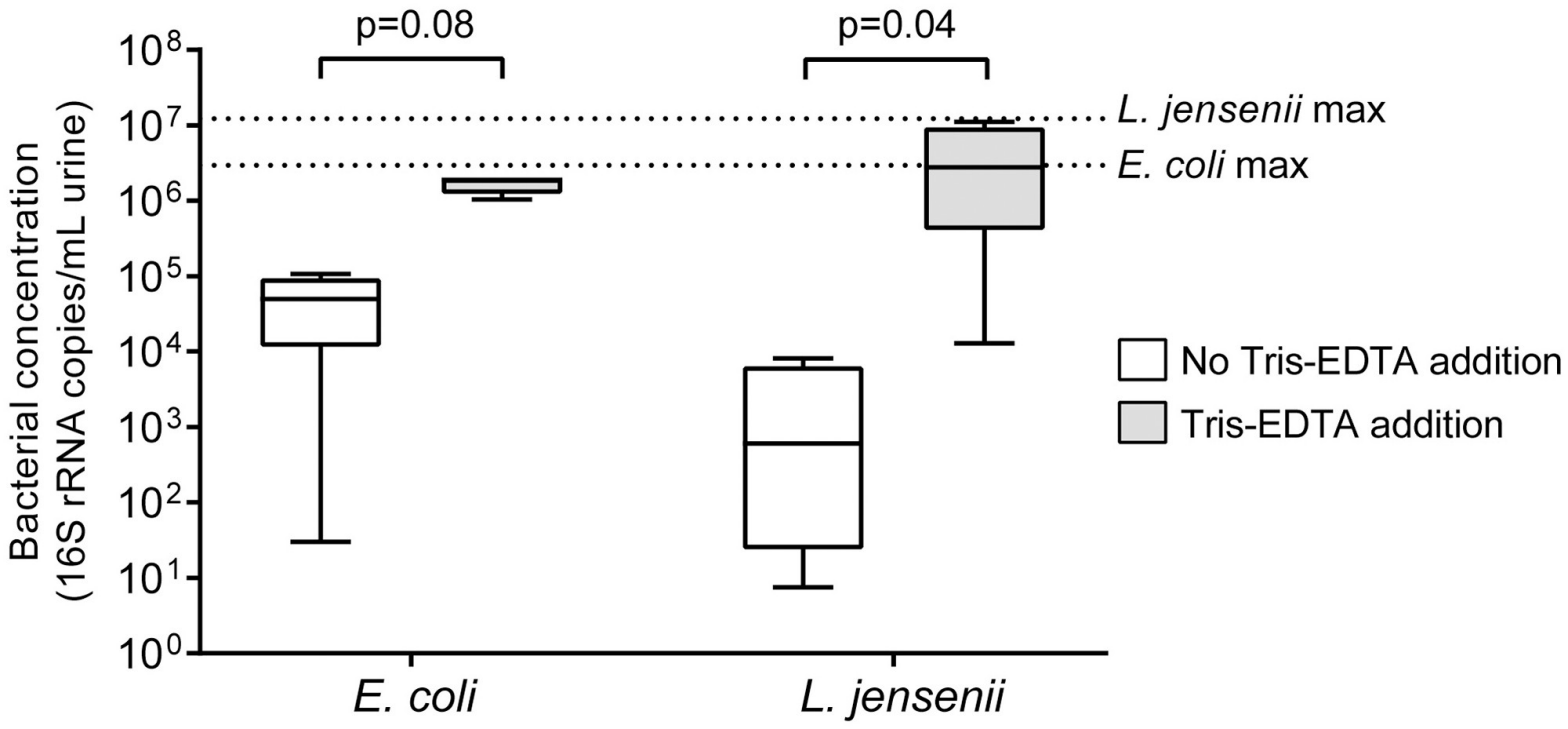
Bacterial DNA recovery from spiked urine with and without Tris-EDTA addition. Data shown as box and whisker plots with whiskers representing maximum and minimum values (16S copies/mL urine). Lines within each box represent median values. Dotted lines represent 100% yield based on spiked positive controls. Mean *Escherichia coli* and *Lactobacillus jensenii* DNA recovery in samples treated with Tris-EDTA were found to be higher than in samples not treated with Tris-EDTA.

*E*. *coli* and *L*. *jensenii* were not detected by qPCR in any non-spiked urine aliquots. *E*. *coli* detection in spiked samples was higher in urine processed with Tris-EDTA compared to samples without (mean 1.9×10^6^ copies/mL with Tris-EDTA vs. 5.0×10^4^ copies/mL, p = 0.08) (Fig 3). Similarly, *L*. *jensenii* detection was significantly higher in urine processed with Tris-EDTA (mean 2.8×10^6^ copies/mL with Tris-EDTA vs. 6.1×10^2^ copies/mL, p = 0.04) (Fig 3). When compared to positive controls of spiked culture, which we considered as 100% efficient, mean percent yield was 57.5% (range 38.3–68.7%) for *E*. *coli* in samples treated with Tris-EDTA compared to 1.8% (range 0–3.5%) in samples not treated with Tris-EDTA. Mean percent yield of *L*. *jensenii* was 33.5% (range 0.11–89.0%) for samples treated with Tris-EDTA compared to 0.02% (range 0–0.06%) for samples not treated with Tris-EDTA.

### Application of optimized approach to clinical samples

**Fig 4:**
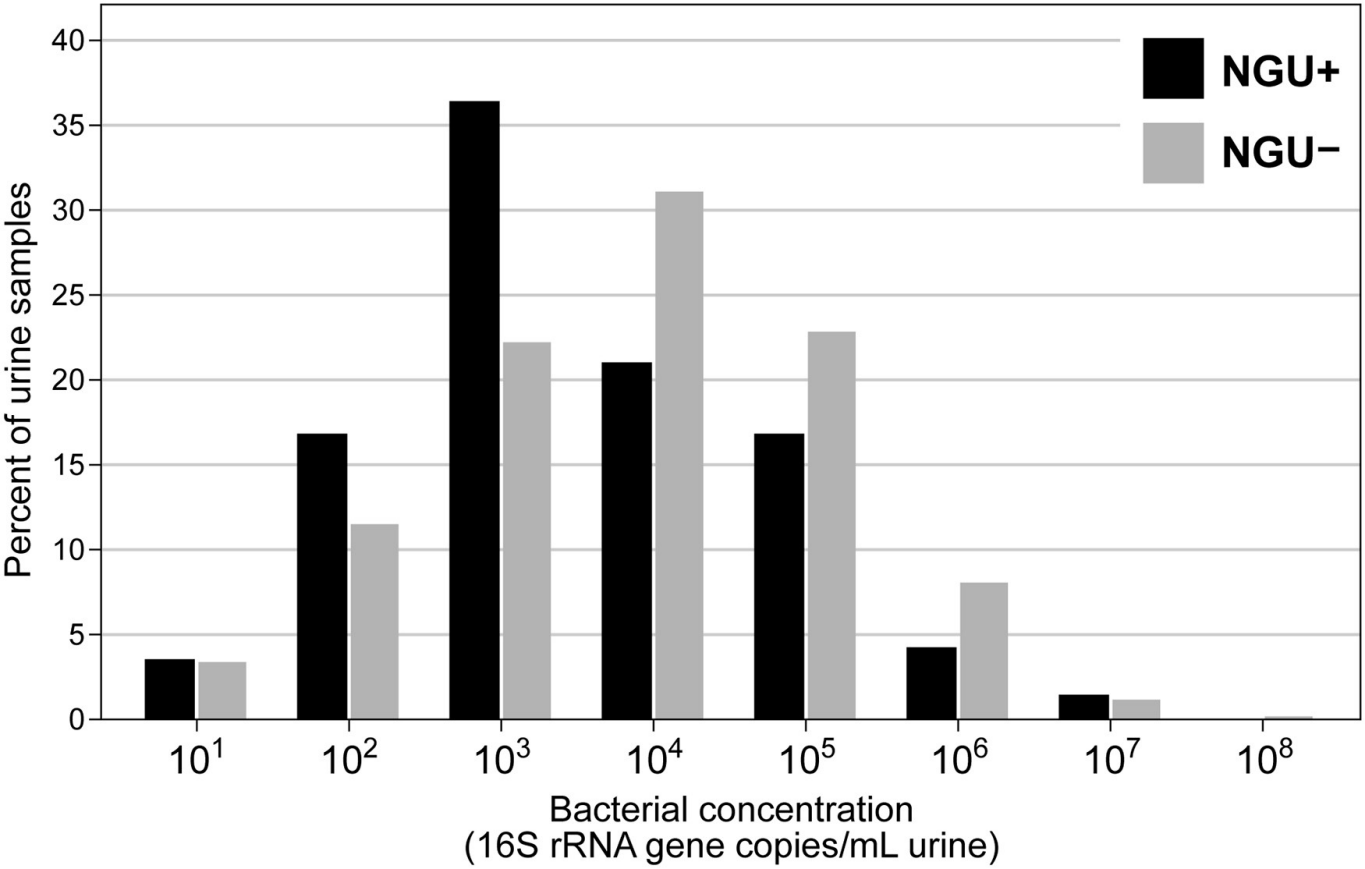
Distribution of bacterial DNA extracted from urine samples treated with 10% v/v Tris-EDTA. Data shown for 955 urine specimens including 143 specimens that were from NGU+ men and 812 specimens that were from NGU- men. An additional 43 samples had bacterial concentrations below the lower limit of detection (16 = NGU+ men and 27 = NGU- men). Bacterial concentrations were significantly lower in men with NGU.

Based on the metrics that were laid out, processing of urine samples with Tris-EDTA was selected as the optimal approach because addition of only 6.7% of the urine volume was required to dissolve crystals in the most concentrated sample tested (Table 1). To account for urine samples with potentially more crystal precipitation and to improve throughput, we increased the amount of buffer added to 10% by volume and applied this protocol to an additional 998 urine specimens. Of 998 specimens, 159 (15.9%) were collected at time points when men had NGU. Bacterial concentrations in all samples ranged from 1.66×10^1^−1.05×10^8^ gene copies/mL urine, with a mean of 7.13×10^5^ gene copies/mL urine and a median of 2.38×10^4^ gene copies/mL urine in 955 samples with concentrations above the lower limit of detection (Fig 4). Buffer controls were included with every batch of DNA extraction from 998 urine samples to determine if there was any contaminant DNA from water or extraction reagents. These negative controls contained 30 mL filtered water and 3 mL Tris-EDTA which were extracted in the same manner as the test urine specimens. Mean 16S rRNA gene copies in these extraction controls was 5.87 copies/mL, which is just above detection threshold. PCR inhibition was detected in 9.1% of samples and was alleviated when DNA was diluted (up to 1:20 dilution) in all but two samples. Bacterial concentrations in urine from NGU positive men ranged from 2.24×10^1^−2.22×10^7^ gene copies/mL urine with a mean of 4.81×10^5^ gene copies/mL urine and a median of 7.30×10^3^ gene copies/mL urine, while bacterial concentrations in urine from NGU negative men ranged from 1.66×10^1^−1.05×10^8^ gene copies/mL urine with a mean of 7.53×10^5^ gene copies/mL urine and a median of 2.77×10^4^ gene copies/mL urine (Fig 4). Bacterial DNA concentrations of urine from men with NGU was 0.61 times lower than concentrations in urine from men without NGU (95% confidence interval = 0.38–0.97, p = 0.04).

## Discussion

Urine is a convenient sample that is useful for defining the genital tract microbiota of men using molecular approaches. Obtaining high concentrations of quality DNA is essential to maximize bacterial detection and identification. Urine is frequently refrigerated or frozen to prevent bacterial overgrowth and to prevent degradation of nucleic acids. However, subjecting urine to low temperatures can lead to precipitation of crystals, which may reduce DNA yields and cause inhibition in downstream molecular applications. Heating urine to dissolve crystals is an approach that is often recommended. For example, the APTIMA Combo 2^®^ Assay protocol for *C*. *trachomatis* and *N*. *gonorrhoeae* testing recommends heating urine to 37°C for up to 5 minutes to dissolve any visible urine precipitates if present (Protocol 502446 Rev. 002, Marlborough, MA). However, heating the urine can potentially lead to bacterial lysis, DNA degradation, or overgrowth of bacteria. It has also been reported that sufficient mixing at room temperature can dissolve crystals formed in urine [53,57], but we found that this approach cannot fully dissolve all crystals in every sample. In this study, we developed an efficient method that can be applied to most urine samples to dissolve crystals prior to urine centrifugation and DNA extraction, resulting in higher bacterial DNA yields and reduced PCR inhibition.

Crystal formation in urine is linked to urine pH, temperature, and concentrations of particular ions. Crystals such as uric acid and amorphous urate crystals are pH dependent and tend to form in acidic urine [47,48], and these crystals typically form pink pellets on centrifugation [47,49,53] which we noted in our study as well (Fig 1). We hypothesized that increasing the pH would result in dissolution of these crystals. We used small volumes of Tris-EDTA to raise urine pH, resulting in crystal dissolution (Fig 1). In contrast, larger volumes of saline and DPBS were required for dissolution of crystals and no increase of pH was noted (Table 1), suggesting that any salutary effect was likely due to dilution alone. Calcium oxalate crystals can form with sufficient Ca^2+^ concentrations in urine and this process is not pH dependent [50]. Saetun et al. found that calcium oxalate and larger amorphous crystals were common in frozen urine samples, and that addition of EDTA to urine pellets reduced pellet size to ~25% of the starting amount [53]. EDTA is a known chelating agent that can remove cations such as Ca^2+^ and Mg^2+^ from solution and may aid in dissolving calcium oxalate crystals by reducing calcium concentrations in urine. Calcium and magnesium ions can also form urate crystals [47], and the chelation of these cations could be important in reversing crystal formation. Moreover, calcium is a known PCR inhibitor that can compete with magnesium to bind to DNA polymerases [58]. Hence, we used Tris-EDTA with the dual purpose of increasing pH and chelating Ca^2+^ and Mg^2+^ ions. We found that the buffer was also effective in reducing PCR inhibition suggesting that chelation of Ca^2+^ and Mg^2+^ ions prior to DNA extraction may be an important step in urine sample processing.

In this study, we processed 998 urine samples with addition of 10% v/v Tris-EDTA and found that bacterial concentrations in these samples ranged from 1.66×10^1^−1.05×10^8^ 16S rRNA gene copies/mL urine with a mean of 7.13×10^5^ gene copies/mL urine and a median of 2.38×10^4^ gene copies/mL urine (Fig 4). All urine in this study was obtained from adult men given that this was a study of NGU in men. Some men were diagnosed with NGU, and samples obtained from these men had a lower concentration of bacterial DNA (Fig 4). Our goal for this study was to optimize DNA extraction of urine samples from men, and hence we did not test any samples from women in this study. This protocol will need to be validated if applied to urine samples collected from women. In addition to measuring total bacterial concentrations, we also monitored PCR inhibition in the samples. Of the 998 samples that we tested, 9.1% exhibited PCR inhibition, although inhibition was overcome with up to a 1:20 dilution of DNA. One potential inhibitor in urine is urea, which can be removed from urine samples by dialysis or ultrafiltration [45], but the approach is not high-throughput and hence not tested in this study.

Extraction efficiency of our optimized approach was determined by spiking urine samples with cultures from a Gram-negative bacterium, *E*. *coli*, and a Gram-positive bacterium, *L*. *jensenii*. DNA recovery from both bacteria was found to be higher in urine aliquots treated with Tris-EDTA than in those without (Fig 3). These data suggest that both Gram-negative and Gram-positive bacteria can be recovered with greater efficiency using our optimized approach. One important limitation is that we did not perform broad-range amplicon PCR and deep sequencing on matched samples to determine if overall bacterial community representation is improved with the addition of Tris-EDTA buffer. Hence, we cannot conclude that this approach will improve yields for all bacteria present in urine. Another limitation of this test was that we compared efficiencies in only four urine samples because urine was collected primarily for questions related to the microbiota and NGU, restricting samples available for optimization of DNA extraction. Despite the limited number of samples tested for extraction efficiency, we showed increased concentrations of bacterial isolate DNA in each of the four samples treated with Tris-EDTA compared to samples that were not treated.

Low DNA yield and PCR inhibition are common problems when extracting bacterial DNA from urine and refrigerating or freezing urine can lead to crystal precipitation that exacerbates these problems. Adding Tris-EDTA to urine samples to increase pH and reduce concentrations of inhibitors prior to DNA extraction is a cost effective, high-throughput solution for improving recovery of bacterial DNA for diagnostic testing and molecular applications aimed at increasing our understanding of bacterial communities in the genital tract.

## Supporting information

S1 Data 16S rRNA gene copies/mL of urine for Figs 2 and 3 and 16S rRNA gene copies with NGU status for Fig 4. (XLSX)
